# A randomized clinical pharmacokinetic trial of Tenofovir in blood, plasma and urine in adults with perfect, moderate and low PrEP adherence: the TARGET study

**DOI:** 10.1186/s12879-017-2593-4

**Published:** 2017-07-14

**Authors:** Tim R. Cressey, Oraphan Siriprakaisil, Virat Klinbuayaem, Justice Quame-Amaglo, Rachel W. Kubiak, Pra-ornsuda Sukrakanchana, Kanchana Than-in-at, Jared Baeten, Wasna Sirirungsi, Ratchada Cressey, Paul K. Drain

**Affiliations:** 1 PHPT-IRD UMI 174, Faculty of Associated Medical Sciences, Department of Medical Technology, Chiang Mai University, 6th Floor, 110 Inthawaroros Road, Muang Chiang Mai, 50200 Thailand; 2 Department of Immunology & Infectious Diseases, Harvard T.H Chan School of Public Health, Boston, MA USA; 3 Department of Molecular & Clinical Pharmacology, University of Liverpool, Liverpool, UK; 4 Sanpatong Hospital, Sanpatong, Chiang Mai, Thailand; 5 Department of Global Health, University of Washington, Seattle, USA; 6 Department of Medicine, University of Washington, Seattle, USA; 7 Department of Epidemiology, University of Washington, Seattle, USA; 8 Division of Medical Technology, Faculty of Associated Medical Sciences, Chiang Mai University, Chiang Mai, Thailand

**Keywords:** HIV, Pre-exposure prophylaxis, Antiretroviral treatment, Tenofovir, Thailand

## Abstract

**Background:**

Tenofovir disoproxil fumarate (TDF) is key component of pre-exposure prophylaxis (PrEP) and antiretroviral therapy (ART) for HIV, but existing tools to monitor drug adherence are often inaccurate. Detection of tenofovir (TFV) in accessible biological samples, such as fingerprick blood, urine or oral fluid samples could be a novel objective measure of recent TDF adherence. To measure TFV concentrations associated with different levels of TDF adherence, we designed a randomized clinical trial to assess the blood, urine and oral fluid concentrations of TFV in adults with perfect, moderate and low drug adherence.

**Methods/design:**

A randomized, open-label, clinical pharmacokinetic study of tenofovir in healthy adult volunteers without HIV or Hepatitis B infection in Thailand. Consenting, eligible participants are randomized (1:1:1) among three groups to receive a controlled number of TDF (300 mg) doses in a combination pill with emtricitabine (FTC, 200 mg) for six weeks. Participants in **Group 1** receive a single TDF/FTC tablet once daily (Perfect adherence); **Group 2** receive a single TDF/FTC tablet 4 times/week (Moderate adherence); and **Group 3** receive a single TDF/FTC tablet 2 times/week (Low adherence). Blood, plasma, urine and oral fluid samples are collected for drug measurement during three study phases: (i) initial 6-week treatment phase; (ii) intensive 24-h blood sampling phase after 6 weeks; (iii) 4-week washout phase. Thirty adults with evaluable pharmacokinetic samples (10 per group) will be enrolled [based on ensuring 25% precision in pharmacokinetic parameter estimates]. Pre-dose drug concentrations during the treatment phase will be descriptive and comparisons between groups performed using a Kruskal-Wallis test. A non-compartmental pharmacokinetic analysis will be performed on the intensive sampling data at Week 7 and the time course of TFV washout in the difference biological matrices will be reported based on the detected concentrations following drug cessation.

**Discussion:**

The results of this randomized trial will define the target concentration thresholds of TFV in blood, urine and oral fluid that can distinguish between different levels of TDF adherence. Such adherence ‘benchmarks’ can be applied to real-time drug testing and novel point-of-care tests to identify individuals with poor PrEP or ART adherence.

**Trial registration:**

ClinicalTrials.gov Identifier NCT03012607.

## Background

Daily oral tenofovir-based pre-exposure prophylaxis (PrEP) has been shown to be safe and effective in reducing the risk of HIV acquisition in adults [1–3]. Truvada®, composed of 300 mg of tenofovir disoproxil fumarate (TDF) and 200 mg of emtricitabine (FTC), is approved for PrEP by the US Food and Drug Administration [4] and is now recommended by the World Health Organization (WHO) for people at substantial risk of HIV infection as part of combination HIV prevention approaches [5]. TDF is also recommended by the WHO as part of first-line antiretroviral therapy (ART) and at the end of 2014 approximately 70% of people on first-line ART were taking a TDF-based regimen [6]. The efficacy of PrEP and ART, however, depend on adequate pill adherence to maintain sufficient drug concentrations [7]. Existing tools to monitor ART and PrEP adherence, such as patient self-reporting [8, 9], pill counting and home-based visits [9–11] have been shown to be ineffective. Electronic pill bottle caps/boxes are more accurate, but their high cost and complexity confine their utility to research settings. Real-time drug testing through simple, low-cost tools to identify poor PrEP or ART adherence would be valuable to help maintain the success of global HIV treatment and prevention programs.

TDF has favorable pharmacokinetic properties for an objective measure of drug adherence. TDF is a prodrug that is rapidly converted to tenofovir (TFV) after oral administration via esterase hydrolysis after absorption [12]. Once inside HIV-infected CD4 T cells, TFV is converted to its active form—TFV-diphosphate (TFV-DP) [12–14]. TFV has a plasma elimination half-life of 17 h and TFV-DP has an intracellular half-life in peripheral blood mononuclear cells (PBMCs) of 4.2 days [15]. An intracellular TFV-DP concentrations of >16 fmol/10^6^ PBMCs was associated with a 90% HIV risk reduction (EC_90_) in HIV acquisition and with ≥4 doses of TDF per week would yield TFV-DP concentrations in PBMCs associated with a 96% HIV risk reduction [7]. While the detection of TFV in plasma and TFV-DP in PBMCs have been proposed as objective measures of recent and cumulative drug adherence, respectively, the use of more accessible biological samples, such as fingerprick blood, urine, or oral fluid samples, would be preferable for real-time drug testing for adherence.

Tenofovir is detectable in whole blood [15, 16], urine [17], and oral fluid [18]. Our hypothesis is that the detection in TFV in a fingerprick blood, urine and/or oral fluid sample provides an indication of recent TDF adherence. However, to date no data are available on the concentration time-course of TFV in these biological matrices among adults with various degrees of TDF adherence. Such data are critical to establish the adherence ‘benchmarks’ for these matrices, which in turn can be applied to interpret drug level monitoring tests.

To address these issues, we have designed a randomized clinical trial to investigate the whole blood, urine and oral fluid pharmacokinetic of TFV in adults with perfect, moderate and low drug adherence. Plasma TFV and intracellular TFV-DP concentrations in PBMCs are also being evaluated to determine the degree to which they correlate with whole blood, urine and oral fluid TFV concentrations and to discriminate longer-term TDF adherence.

## Methods/design

The study is a randomized, open-label, pharmacokinetic study in healthy adult volunteers without HIV or Hepatitis B infection. The clinical study is being conducted in Sanpatong Hospital in Chiang Mai, Thailand, with the measurement of drug concentrations being performed at the Faculty of Associated Medical Sciences (AMS) at Chiang Mai University. All participants are screened for eligibility and asked to provide written informed consent before study participation.

Participant screening is performed within 14 days of enrollment. At screening, a detailed background demographic and health questionnaire is administered and blood is drawn for baseline assessments including complete blood count, HIV antibody testing, Hepatitis B surface Ag testing, renal and liver function tests. The results of the clinical examination and laboratory assessments are reviewed for the inclusion/exclusion criteria described below:

### Inclusion criteria

- Willing/able to provided written informed consent
- Age ≥ 18 and <50 years old
- HIV and Hepatitis B surface Ag negative
- Normal renal function (estimated glomerular filtration rate (GFR) >60 mL/min by the Cockcroft-Gault equation)

### Exclusion criteria

- Pregnant female
- Any significant lab abnormality of neutrophil count, hemoglobin, platelets, aspartate aminotransferase (AST), or alanine aminotransferase (ALT) (Defined as Grade ≥ 3 by Division of AIDS Table for Grading the Severity of Adult and Pediatric Adverse Events, Version 2.0, Nov. 2014)
- History of using PrEP or eligible to receive PrEP
- Any clinically significant diseases or clinically significant findings during the screening medical history or physical examination that, in the investigator’s opinion, might compromise participation in this study
- Any concurrent participation in another clinical trial

### Randomization

At enrollment, eligible participants are randomized (1:1:1) among three groups to receive a controlled number of doses of TDF/FTC (300/200 mg) in a combination pill (Truvada®, Gilead Sciences), see Table 1. Participants in **Group 1** receive a single TDF/FTC tablet once daily for 6 weeks, representing ‘Perfect’ adherence (reference group). Participants in **Group 2** receive a single TDF/FTC tablet 4 times/week for 6 weeks, representing ‘Moderate’ adherence; and participants in **Group 3** receive a single TDF/FTC tablet 2 times/week for 6 weeks, representing ‘Low’ adherence. A total of 30 adults with evaluable pharmacokinetic (PK) samples (10 per group) will be enrolled.

**Table 1:** Summary of the three randomization groups and the controlled levels of drug adherence

Randomized Groups	Level of Adherence	Controlled Intake of Truvada
Group 1 (*n* = 10)	‘Perfect’ Adherence (Daily)	Single tablet of Truvada® once daily for 6 weeks
Group 2 (*n* = 10)	‘Moderate’ Adherence (4 times/week)	Single tablet of Truvada® 4 times per week (Monday, Wednesday, Friday and Saturday) for 6 weeks
Group 3 (*n* = 10)	‘Poor’ Adherence (2 times/week)	Single tablet of Truvada® 2 times per week (Monday, Thursday) for 6 weeks

**Fig. 1:**
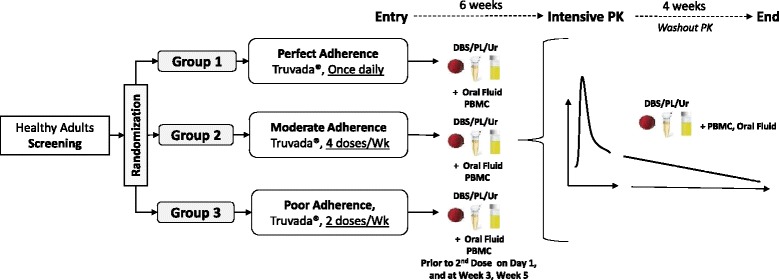
Study schema

The 6-week treatment period was chosen in order to ensure concentrations of TFV in the different matrices, e.g. blood, plasma, urine, oral fluid, PBMCs, mimic the conditions expected in patients with chronic dosing. The 4-week washout period was based on the reported TFV half-life in the various biological samples under study. The study schema summarizing the treatment/washout periods and specimen sampling schedules are depicted in Fig. 1.

### Initial 6-week treatment phase

After randomization, all participants start Truvada® in the morning of the entry visit. During the next 6 weeks of controlled drug intake, pre-dose specimens are collected before the 2nd dose of TDF/FTC, and at Weeks 3 and 5 (both Monday morning, Table 2).

**Table 2:** Summary of the clinical visits and laboratory testing for all 3 study groups

	Screening (≤14 days to Entry)	Entry	Prior 2nd Dose	Weeks 3 & 5	Week 7 (Monday/ Tuesday) PK visit	Weeks 7 (Wednesday Thursday, Friday, Saturday, & Sunday) Washout	Week 8 (Monday & Thursday) Washout	Week 9 Washout	Week 10 Start/End Washout
Informed Consent	x								
Physical Exam	x	x			x				
Medical History		x	x	x	x	x	x	x	x
Study Drug Dispensing		x	x	x	x				
Assess medication side effects			x	x	x	x	x	x	x
Adherence to Medication			x	x	x				
HIV Serology (2 ml)	x								
Hematology^a^ (2 mL)	x								
Chemistry^b^ (2 mL)	x			x	x				x^C^
Hepatitis B surface Ag (1 mL)	x								
DBS/Plasma PK Sample (3-5 mL)			x	x	x	x	x	x	x
Fingerstick (DBS) (0.1 mL)			x	x	x	x	x	x	x
Spot Urine Sample (5–10 mL)			x	x	x	x	x	x	x
Collection of 24-h Urine					x	x			
Oral Fluid			x	x	x	x^e^			
PBMC (10 mL)			x	x	x		x^d^	x	x
Store plasma/EDTA Cell Pellet (3 mL)	X								
Total Blood Vol. (mL)	10 mL	0 mL	15 mL	17 mL	39 mL	25 mL	20 mL	15 mL	32 mL

^a^Hematology: hemoglobin, hematocrit, RBC, MCV, WBC and differential, platelets

^b^Chemistry: AST, ALT, total bilirubin, BUN, creatinine; electrolytes: sodium, potassium, chloride, bicarbonate, calcium, phosphate

^c^Only at End of Week 10

^d^Only On Monday of Week 8

^e^Only on Wednesday, Thursday, Friday of Week 7

### Intensive 24-h PK blood sampling phase

On the Monday morning of Week 7, a pre-dose blood sample is drawn and a single Truvada® tablet is administered by the study team on an empty stomach (with at least 6 h fast beforehand). This is the last dose of Truvada® for all subjects. Venous blood samples are then collected at 1, 2, 4, 6, 8, 10, 12 and 24 h post-dose. At each time point, a venous blood dried blood spot (DBS, 5 spots) and fingerstick DBS (2 spots) are collected. Oral fluid samples are collected at pre-dose, 1, 4, 12 and 24 h post-dose. Urine samples are collected pre-dose and over the intervals of 0–4 h, 4–8 h, 8–12 h, and 12–24 h. PBMCs are only collected pre-dose. Renal and liver function tests are performed for safety evaluations.

### 4-week wash-out phase

After the intensive PK sampling is complete, subjects return to the clinic once a day for the remaining 5 days of Week 7 for blood, urine (24-h) and oral fluid specimen collection (Table 2).

Blood and urine are collected on Monday and Thursday of Week 8, and then on Monday morning of Weeks 9 and 10. Blood and urine are collected and a final blood chemistry is performed at the last study visit (end of Week 10) on the following Monday morning.

### Assessment of study treatment adherence

We are utilizing several monitoring tools to ensure strict compliance to the assigned randomized adherence groups. Directly Observed Therapy (DOT) is performed for each study drug intake for all subjects. Subjects are asked to come to the clinic each morning they are required to take the study treatment. At weekends (specifically for Groups 1 and 2), DOT is remotely implemented to document each drug administration, either via video call or mobile phone application. To complement DOT, each subject is also provided with an electronic “Wisepill” box (https://www.wisepill.com), whereby mobile phone and internet technologies send a message in real-time to a central database each time the subject opens the pill box. The study team closely monitors the records of the Wisepill box use and is able to cross reference results with the DOT records.

### Measurement of drug concentrations

Samples collected for drug measurement are stored at −70 °C to −80 °C until analysis. Drug concentrations are measured using validated liquid chromatography mass spectrometry (LC-MS/MS) assays. All methods are being validated in accordance with the Clinical Pharmacology Quality Assurance and Quality Control Program (CPQA) method validation guidelines [19], which are required for analyzing study specimens within the Division of AIDS (DAIDS) HIV clinical trial networks (based on the US Food and Drug Administration Guidance for Industry Bioanalytical Method Validation [20]). This laboratory participates in two international External Quality Control (EQC) programs for quantification of antiretroviral drugs: (i) the HIV/AIDS Clinical Pharmacology Quality Assurance program from the University at Buffalo, NY, which performs standardized inter-laboratory testing twice a year [21], and (ii) ASQUALAB Quality Control program, France (http://www.asqualab.com/).

### Sample size calculation

The sample size is based on ensuring precision in pharmacokinetic parameter estimates to accurately describe the pharmacokinetics of TFV. Using the plasma pharmacokinetic parameters as a reference value, the precision required is a mean TFV plasma AUC_0-24h_ at steady state within ±25% of the true population mean. The mean (± standard deviation) TFV AUC_0-24h_ in HIV-infected adults is 3.32 ± 1.37 μg.hr./mL [coefficient of variation (CV) of 41.2%] following multiple doses of 300 mg once daily [13]. Assuming the true population mean plasma AUC_0-24h_ at steady state is the same for healthy subjects, the sample mean must fall within ±0.83 μg.hr./mL of the true mean to ensure 25% precision. Based on this variability, at the standard dose and assuming average AUC results are normally distributed, to be 90% confident that the plasma TFV AUC_0-24h_ sample mean is within 0.83 μg.hr./mL of the true mean, a sample size of 10 participants is required for each of the 3 groups, making 30 participants in total. Subjects who do not complete the study will be replaced.

### Statistical analysis

The primary endpoints are descriptive pharmacokinetics endpoints. A Kruskal-Wallis test will be used to compare basic demographic variables between the three groups. A non-compartmental pharmacokinetic analysis will be performed on the TFV concentration data generated from the intensive blood samplings at Week 7. Pharmacokinetic parameters in plasma and blood (venous and fingerstick) will include: area-under-the-curve (AUCτ), maximum concentration (C_max_), time to C_max_ (T_max_), apparent clearance (CL/F), apparent volume of distribution (V/F), and minimum concentration (C_min_). AUCτ will be determined using the linear trapezoidal method. C_max_, C_min_ and T_max_ will be taken directly from the observed concentration-time data. The terminal slope (λz) will be determined from the log-linear portion of the curve, and the half-life will be calculated as Log_10_ (2)/λz. Total body clearance (CL/F) will be calculated using Dose/AUC_0-τ_. The fraction of TFV excreted unchanged in the urine (Fe_TFV_) will be determined as the total amount of TFV excreted during the urine collection period following cessation of RDF divided by the dose. The renal TFV clearance (CLR) will be calculated by multiplying Fe_TFV_ and CL/F. We will calculate median (range), means (standard deviations), and geometric means with 95% confidence intervals for each PK parameter.

We will compare pre-dose TFV concentrations in specimens collected during the treatment lead-in period (i.e. before 2nd dose of TDF/FTC, and at Weeks 3, 5 and 7) using a Kruskal-Wallis test. We will report the time course of TFV washout in blood, plasma, urine and oral fluid based on the detected drug concentrations following drug cessation. We will perform non-pharmacokinetic statistical analyses using Stata (Version 10.0, StataCorp LP, Texas, USA).

## Discussion

The efficacy to TFV-based PrEP and ART are highly dependent on adequate medication adherence to maintain sufficient drug concentrations. Improvements in real-time drug testing and novel point-of-care tools are needed to help improve monitoring of drug adherence. Detecting TFV concentrations in easily accessible biological specimens, such as fingerprick blood, urine or oral fluid, may act as a simple objective measure of recent TDF adherence. The objective of this trial is to characterize the pharmacokinetics of TFV among healthy volunteers with controlled drug adherence (perfect, moderate, and low) in fingerprick blood, urine and oral fluid. The information generated from this clinical study will be critical to determine if TFV concentrations in these biological specimens can distinguish between different levels of TDF adherence.

Moreover, these data are highly relevant to research towards the development of inexpensive, rapid, point-of-care drug measurement tests for monitoring medication adherence. The clinical data on the pharmacokinetics of TFV (and/or FTC) in these biological specimens with respect to controlled adherence will help define the target thresholds, or adherence ‘benchmarks’, that can facilitate clinical interpretation of real-time testing and novel point-of-care tests. The introduction of scalable TFV-based point-of-care tests may allow rapid identification of people struggling with PrEP or ART adherence, in order to develop and implement targeted adherence interventions. Improving adherence to both PrEP and ART will help reduce and prevent HIV transmission, while also preserving the use an important drug in the efforts to end the HIV/AIDS epidemic.

## Abbreviations

ALT: alanine aminotransferase
AMS: Faculty of Associated Medical Sciences
ART: antiretroviral therapy
AST: aspartate aminotransferase
AUC: area-under-the-curve
CI: confidence interval
CL/F: apparent clearance
C_max_: maximum concentration
C_min_: minimum concentration
CPQA: Clinical Pharmacology Quality Assurance
DAIDS: Division of AIDS
DBS: dried blood spots
DOT: Directly Observed Therapy
Fe: fraction of drug excreted unchanged in the urine
FTC: Emtricitabine; GFR, Glomerular filtration rate
NIH: National Institutes of Health
PBMCs: Peripheral blood mononuclear cells
PK: Pharmacokinetics
PrEP: Pre-exposure prophylaxis
TARGET study: Tenofovir Adherence to Rapidly Guide and Evaluate PrEP and HIV Therapy
TDF: tenofovir disoproxil fumarate
TFV: Tenofovir
TFV-DP: TFV-diphosphate
T_max_: time to maximum concentration
V/F: apparent volume of distribution
WHO: World Health Organization
λz: terminal slope
